# Impact of *Lacticaseibacillus rhamnosus* GG on the Emulsion Stability of Raw Milk

**DOI:** 10.3390/foods10050991

**Published:** 2021-05-01

**Authors:** Raphael Dos Santos Morais, Nicolas Louvet, Frederic Borges, Dominique Dumas, Loubiana Cvetkovska-Ben Mohamed, Sarah Barrau, Joël Scher, Claire Gaiani, Jennifer Burgain

**Affiliations:** 1 LIBio, Université de Lorraine, F-54000 Nancy, France; raphael.dos-santos-morais@univ-lorraine.fr (R.D.S.M.); frederic.borges@univ-lorraine.fr (F.B.); loubiana.cvetkovska-ben-mohamed@univ-lorraine.fr (L.C.-B.M.); sarah.barrau@univ-lorraine.fr (S.B.); joel.scher@univ-lorraine.fr (J.S.); claire.gaiani@univ-lorraine.fr (C.G.); 2 CNRS, LEMTA, Université de Lorraine, F-54000 Nancy, France; nicolas.louvet@univ-lorraine.fr; 3 CNRS, IMoPA, Université de Lorraine, UMS2008 IBSLor-CNRS-UL-INSERM, Plateforme d’Imagerie et de Biophysique Cellulaire de Nancy (PTIBC IBISA-NANCY), F-54000 Nancy, France; dominique.dumas@univ-lorraine.fr; 4 Institut Universitaire de France (IUF), 75231 Paris, France

**Keywords:** raw milk, fat globule, creaming, flocculation, lactic acid bacteria, *Lacticaseibacillus rhamnosus* GG (LGG), microbial distribution, confocal laser scanning microscopy (CLSM)

## Abstract

Lactic acid bacteria (LAB) have been studied for several decades to understand and determine their mechanism and interaction within the matrix into which they are introduced. This study aimed to determine the spatial distribution of *Lacticaseibacillus rhamnosus* GG (LGG) in a dairy matrix and to decipher its behaviour towards milk components, especially fat globules. Two strains of this widely studied bacterium with expected probiotic effects were used: LGG WT with pili on the cell surface and its pili-depleted mutant—LGG Δ*spaCBA*—in order to determine the involvement of these filamentous proteins. In this work, it was shown that LGG Δ*spaCBA* was able to limit creaming with a greater impact than the wild-type counterpart. Moreover, confocal imaging evidenced a preferential microbial distribution as aggregates for LGG WT, while the pili-depleted strain tended to be homogenously distributed and found as individual chains. The observed differences in creaming are attributed to the indirect implication of SpaCBA pili. Indeed, the bacteria-to-bacteria interaction surpassed the bacteria-to-matrix interaction, reducing the bacterial surface exposed to raw milk. Conversely, LGG Δ*spaCBA* may form a physical barrier responsible for preventing milk fat globules from rising to the surface.

## 1. Introduction

Milk is used to design secondary products, such as yogurts or cheese. It is a complex colloidal system whose main constituents are water, fat in the form of fat globules, proteins, lactose and minerals [1]. Milk usually undergoes technological processes, such as homogenization or heat treatment in order to ensure its conservation, since the system is unstable over time [1]. Milk is an oil-in-water emulsion composed of 90% water presenting a supramolecular organization with fat globules (diameters ranging from 1 to 10 µm) surrounded by the milk fat globule membrane (MFGM), which has a high polar lipid and protein content [2]. This emulsion may become unstable due to physical instability, i.e., a change in the spatial arrangement or size distribution of the fat globules, such as creaming, flocculation or coalescence [3,4]. Flocculation is defined as a reversible aggregation mechanism that occurs when droplets combine as a result of unbalanced attractive and repulsive forces [5]. On the other hand, coalescence is an irreversible mechanism whereby fat globules merge together. Natural creaming occurs when the native fat globules present in non-agitated raw milk rise to the surface and form a concentrated layer at the top of the sample, with no change in the droplet size distribution [6]. Creaming is a reversible phenomenon, as the original uniform distribution of droplets can usually be obtained by slow mixing. For low concentrations, the creaming process can usually be described by Stokes’ law [5,7]. The creaming rate can be reduced by decreasing the radius of the fat globule, increasing the continuous phase viscosity and/or decreasing the difference in density between the two phases. Another important phenomenon is the diffusion of fat globules which counteract creaming. Small fat globules rise to the surface very slowly under gravity, but diffuse rapidly. Walstra and Oortwijn [7] used Stokes’ law to predict creaming and observed some deviations, with actual creaming being slower than predicted in theory, particularly when fat globules were very small or when the fat content was high [8]. In raw milk, flocculation, coalescence and creaming are the three synergistically occurring phenomena [9]. Fat globule size and density are the major factors affecting creaming, and homogenization is among the major processes used to control the creaming rate. During homogenization, fat globules are disrupted mechanically, and their sizes are reduced to less than 1 μm. This increases the surface area, and the original MFGM is insufficient to stabilize the newly formed surfaces completely. The fat globules are thus partially stabilized by the adsorption of milk proteins [7,10]. Coalescence gradually results in the separation of the lipid phase from the serum phase and is irreversible. Coalescence requires the rupture of the stabilizing membrane (MFGM) at the oil–water interface, but this occurs only when the continuous phase film between the droplets has thinned to a certain critical thickness [11,12,13,14]. During the production of fermented dairy products, lactic acid bacteria (LAB) are initially added to the milk, in order to catabolize lactose. This produces lactic acid, which leads to milk acidification. The initial spatial distribution of the bacteria in milk is crucial for the subsequent bacterial location inside the curd [15]. It has been reported that in cheeses, bacteria tend to be located near the fat droplets or near the fat–protein interphase [16]. Bacterial distribution is driven by the bacteria–matrix interactions and microbial surface properties, such as surface charge and hydrophobicity, which are able to modulate these interactions [17,18,19]. The presence of specific components, such as adhesins, is also crucial for bacterial interaction with the dairy matrix. *Lactococcus lactis* strains were surface engineered to investigate the effect of cell surface components on textural properties [20]. Their distributions in cheese curd were also studied [21]. Recently, it has been shown that the SpaCBA pili of *Lacticaseibacillus rhamnosus* GG (LGG) [22] were able to specifically interact with β-lactoglobulin [23,24] and the MFGM [25]. The spatial distribution of bacteria in fermented dairy products has mainly been studied in the final product, but there is limited knowledge about the phenomena occurring in the very first steps of the fermentation process, notably concerning the distribution of bacteria. In the present study, the role of the microbial surface composition on bacterial distribution in raw milk was evaluated using the well-documented LGG. The role played by SpaCBA pili was subsequently investigated. Since raw milk is known to be naturally unstable, the role of LGG on emulsion stability was also evaluated.

## 2. Materials and Methods

### 2.1. Material

Raw bovine milk was provided by a local farm (experimental estate of La Bouzule, ENSAIA, Laneuvelotte, France), and was stored at 4 °C with no additional processing carried out before use.

### 2.2. Bacterial Strains and Culture Conditions

The *Lacticaseibacillus rhamnosus* ATCC53103 model strain (LGG wild-type, “LGG WT”) and a derivative mutant strain CMPG 5357 (Δ*spaCBA*:Tc^R^), impaired in pili synthesis [26], were used in this study. All strains were pre-cultivated at 37 °C overnight in 10 mL of MRS (de Man, Rogosa and Sharpe) broth (BIOKAR Diagnostics, Solabia Group, Allonne, France) inoculated with 100 µL of frozen cultures previously stored at −80 °C. On the next day, 100 µL of the pre-cultures were used to inoculate 10 mL of MRS broth, and incubation was conducted at 37 °C until the optical density at 595 nm reached 0.8. Bacterial suspensions were finally centrifuged at 3000× g, 10 min, 25 °C), and the resulting cell pellets were used for incorporation into milk.

### 2.3. Preparation of Milk-Containing LGG Samples

An amount of 10 mL of raw milk was taken and kept at 25 °C for one to two hours in order to reach this temperature before being added to cell pellets. The 10 mL of raw milk was then added to the cell pellets, and the bacteria were gently re-suspended in milk. The concentration of bacterial cells in the samples was 10^8^ CFU/mL. The emulsion stability and spatial microbial distribution were immediately evaluated.

### 2.4. Emulsion Stability

The stability of the emulsion over time was monitored using a Turbiscan classic MA2000 (Formulaction, Toulouse, France). This device measures the turbidity of a liquid sample based on the principle of multiple light scattering using a near-infrared laser source (850 nm). The major destabilizations that can be observed using this device are particle migration (creaming, sedimentation and clarification) and particle size variations (flocculation or coalescence) [27,28]. The sample was placed in a cylindrical glass cell and scanned over its full height every 40 μm. Two optical detectors received the light flux: a transmission detector receiving the light transmitted through the sample (in the same direction as the incident light source) and a backscatter detector retrieving the light reflected by the sample (45°) from the incident ray.

Two resulting curves can be plotted as a function of the sample height: one presenting the transmission light flux (in %), which accounts for the transparency of the sample, while the second, useful for the study of liquid milk matrices, provides information on the opacity of a sample; this is the backscattered light flux (%). The cylindrical glass cell was filled with 7 mL of sample, and data were acquired at 25 ± 1 °C every 2 min for the first hour, and then every 10 min for two additional hours. The typical graph summarizing the measurements is presented in Figure S1. The *X*-axis represents the height of the sample (bottom to top, from left to right in mm), and the *Y*-axis represents the relative backscattering signal (%) (backscattering signal from which the signal at initial time was subtracted). Data processing was performed using the MA2000 software Turbisoft version 1.2.2 (Formulaction) to determine the rates of natural milk phenomena, such as creaming rate and flocculation.

### 2.5. Microbial Distribution in the Milk Matrix

One millilitre of milk-containing LGG WT or LGG Δ*spaCBA* was labelled with the LIVE/DEAD BacLight viability kit (1:200 *v*/*v*; the LIVE/DEAD BacLight viability kit was prepared according to the procedure described for the L13152 kit by ThermoFisher Scientific, Waltham, MA, USA). Lipids were stained using Nile red (5:200 *v*/*v*; Sigma-Aldrich, St. Louis, MO, USA; the solution was prepared in PEG 200 according to [29]. Two hundred microlitres of suspension-containing LGG were placed on chambered glass slides (Nunc Lab-Tek, ThermoFisher Scientific, Waltham, MA, USA). Confocal laser scanning microscopy (CLSM) images were taken using a Leica TCS SP5-X-AOBS confocal laser scanning microscope (Leica Microsystems CMS GmbH, Mannheim, Germany) equipped with WLL lasers. The objective lens used was a HCX PL APO CS 100 × 1.40 (oil immersion). Image acquisition was performed in sequential mode with the following excitation and emission wavelengths: (1) λ_ex1_ = 488 nm and λ_em1_ = 490–555 nm; (2) λ_ex2_ = 488 nm and λ_em2_ = 560–600 nm. The images were processed and exported using LAS X software, version 3.4.2.18368. Three independent repetitions were performed, and approximately 20 representative images were acquired for each repetition. Image sequences were acquired at frequency f = 2 Hz in order to investigate the movements of LGG in the milk over time. The bacterial dynamic was illustrated by calculating the cumulative area covered by the bacteria as a function of time. For each image, the area of all the bacteria was measured first by applying a threshold function and then an edge-detection filter. The cumulative area was calculated over time and ensemble average, and finally normalized according to the initial area:

(1) $$A^{*}\left(t\right)=\frac{1}{A\left(t_{0}\right)}\underset{i=1,\ldots ,N}{\cup }A\left(t_{i}\right)$$

where N is the number of images in the sequence and $A\left(t_{i}\right)$ the bacteria area for the image at time $t_{i}$, $t_{0}$ being the first image analysed in the sequence.

## 3. Results

### 3.1. Raw Milk Stability

**Figure 1:**
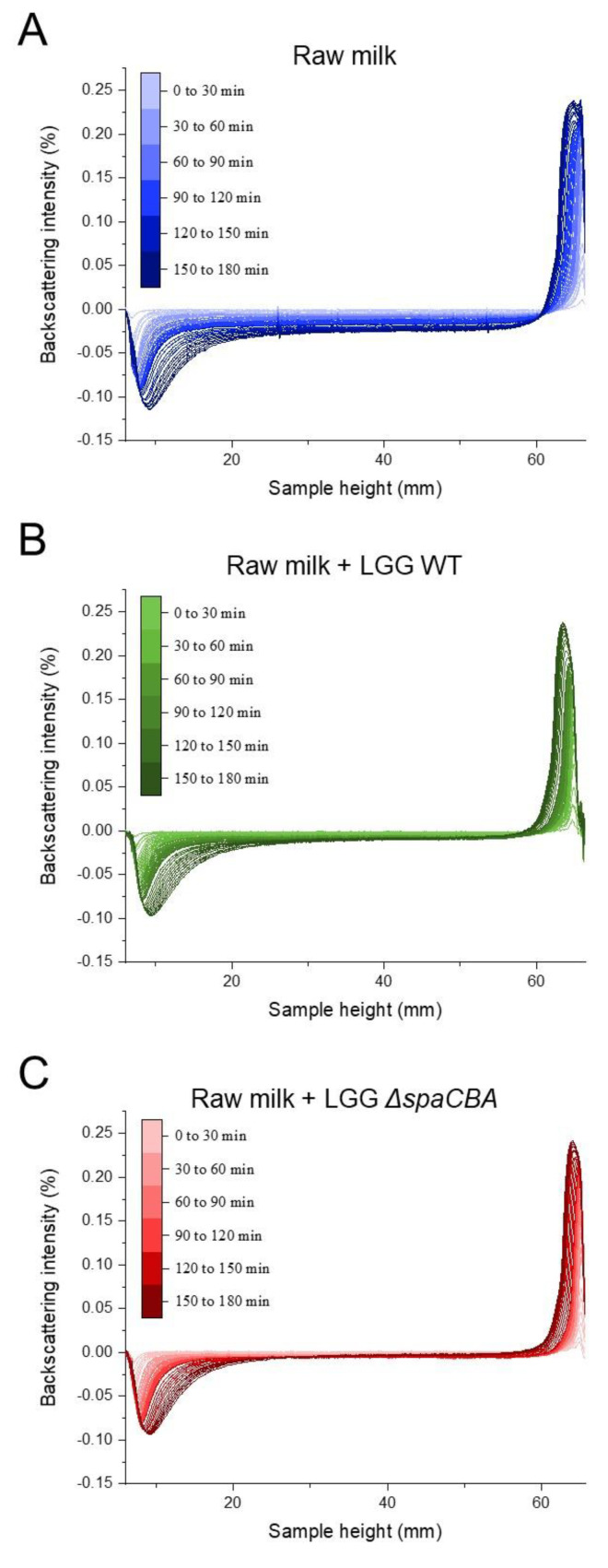
Backscattering intensity vs. sample height of (**A**) raw milk, (**B**) raw milk + LGG WT and (**C**) raw milk + LGG Δ*spaCBA*.

**Figure 2:**
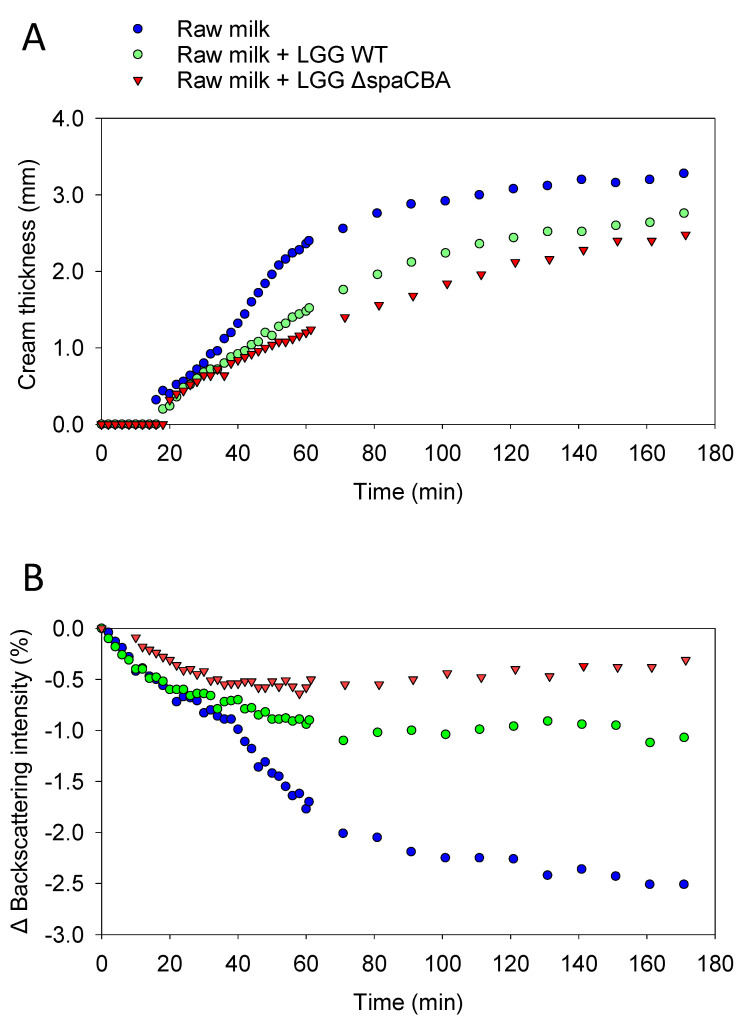
(**A**) Thickness of the cream layer obtained on the top of the sample as a function of time. (**B**) Backscattering intensity in the centre of the cylinder (35 mm) as a function of time. Raw milk (blue circle), raw milk-containing LGG WT (green circle) and raw milk-containing LGG Δ*spaCBA* (red triangle).

Before investigating the effect of the bacteria on raw milk, a control sample was taken to determine whether during these 3 h of analysis, the fat globules mainly tended to flocculate or coalesce. A first analysis of a sample of raw milk was carried out, and then at the end of the analysis, the sample was gently mixed and analysed for a second time by Turbiscan. The two results obtained were identical, meaning that the fatty globules did not have time to merge with one another; the phenomenon was reversible by mixing; and it, therefore, mainly involved flocculation (Figure S2). The results of this control analysis confirmed that flocculation is the phenomenon highlighted in the present study and that no coalescence was observed. Next, the evolution of the emulsion without bacteria was observed (Figure 1A), and the evolution with added bacteria was also analysed over time (Figure 1B,C). In the absence of bacteria, the backscattering intensity increased on the top of the sample (60–70 mm), which is the result of fat globule accumulation due to creaming. On the contrary, at the bottom of the sample (i.e., 0–10 mm), a negative peak reflected a lower concentration of fat globules, indicating clarification. In the middle of the sample (i.e., 10–60 mm), the signal decrease was typical of flocculation (Figure 1A). With regard to the addition of LGG to raw milk (Figure 1B,C), it can be observed that the addition of bacteria to raw milk tends to attenuate natural creaming by limiting the rising of fat globules to the surface. The presence of either LGG WT or LGG Δ*spaCBA* is associated with a modification of cream thickness at the end of the analysis. It should be noted that fat globule flocculation seems to be strongly affected since the decrease in the backscattering signal in the middle of the sample was less pronounced in raw milk-containing LGG WT, and was even more pronounced for LGG Δ*spaCBA* compared to raw milk without bacteria. A quantitative analysis was carried out on the basis of the results in Figure 1 (Figure 2 and Table 1). This analysis concerned the thicknesses of the cream layer at the top of the sample, the creaming rate (Figure 2A), and the backscattering intensity in the middle of the sample, corresponding to the flocculation phenomenon (Figure 2B). Concerning the thickness of the cream layer and the creaming rate, the accumulation of fat globules over time occurred in the upper phase of the sample. There were differences in the creaming rates of raw milk and raw milk-containing LGG. The change in cream layer thickness over time can be described by a three-step mechanism. First, from 0 to 20 min, there was no change in the thickness of the cream layer. Second, an exponential increase was observed. This step was impacted by the presence of LGG WT, and even more so by LGG Δ*spaCBA*. Third, a plateau was reached in the case of raw milk in the absence of bacteria. After 3 h, the final cream layer presented a thickness of 3.3 mm for raw milk, and only 2.8 mm and 2.5 mm for LGG WT and LGG Δ*spaCBA*, respectively (Figure 2 and Table 1). This difference in thickness could be explained by the retention of fat globules within the matrix, with the bacteria being able to form a barrier to their dynamics. Plotting the cream layer thickness over time provides a global view of the movements of fat globules rising to the surface. The bacteria mainly impacted the creaming rate calculable in the exponential step. The creaming rate of the raw milk (~60 µm/min) decreased in the presence of LGG WT (~30 µm/min) and to an even greater extent in the presence of LGG Δ*spaCBA* (~20 µm/min) (Table 1). The creaming rate of this sample was twice as fast as for milk-containing LGG WT and more than three times faster than for milk-containing LGG Δ*spaCBA*. Notably, the presence of bacteria in raw milk reduced both the creaming rate and cream thickness.

Flocculation leads to an increase in particle size. The induced variation in particle size can be studied using the Turbiscan, since it leads to a reduction in backscattering over the entire height of the sample [27,28]. Raw milk presents fat globule destabilization over time, with this phenomenon evidenced here by the decrease in backscattering intensity in the central part of the cylinder (Figure 1A). In the presence of bacteria, this phenomenon is clearly affected and less noticeable. The variations in backscattering intensity over time for raw milk and raw milk-containing LGG WT or LGG Δ*spaCBA* are presented in Figure 2B. After 3 h of acquisition, for raw milk, a decrease of ~−2.5% was observed and ~−1% in the presence of LGG WT. Notably, a reduction of only ~−0.5% was detectable for LGG Δ*spaCBA,* revealing a marked decline in flocculation. Bacteria seem to impact fat globule flocculation, and it can be hypothesized that the presence of bacteria physically limits the interaction between fat globules, which is crucial for flocculation and the putative ensuing coalescence, which might not be observable within the timescale analysed here. It should be noted that the reduction in flocculation was more pronounced for LGG Δ*spaCBA* than for LGG WT. The decline in the backscattering percentage highlights the changes in fat globule size over time. The lower this percentage, the greater the flocculation of the fatty globules, and, therefore, the larger their size without coalescing. These analyses reveal that in the samples, the bacteria do not increase the size of the fat globules drastically in comparison to the samples of raw milk without bacteria, which destabilize more significantly over time. In order to validate these observations on independent raw milk, another batch sample was analysed in the same way (Figure S3 and Table S1). Although the absolute values were different and attributed to raw milk composition at a precise moment, the relative evolution was identical and only depended on the strain added to the sample. In sum, the strains used in the present study are, therefore, able to influence natural creaming by reducing fat globule flocculation and the ensuing creaming rate and cream layer. All these different results provide substantial information about the role of microbial surface components. Since the difference between LGG WT and LGG Δ*spaCBA* is the absence of SpaCBA pili, bacteria-to-bacteria interactions as well as bacteria–matrix interactions impact the emulsion structure. Contrary to LGG WT, LGG Δ*spaCBA* might expose more surface molecules liable to interact with fat globule components. Bacterial pili might induce steric hindrance due to their size, thus reducing the accessibility to the other surface molecules, but this requires further investigations. It, therefore, appeared essential to study bacterial behaviour at the microscale using microscopy to explain the changes in the emulsion over time.

### 3.2. Microbial Distribution in Raw Milk

**Figure 3:**
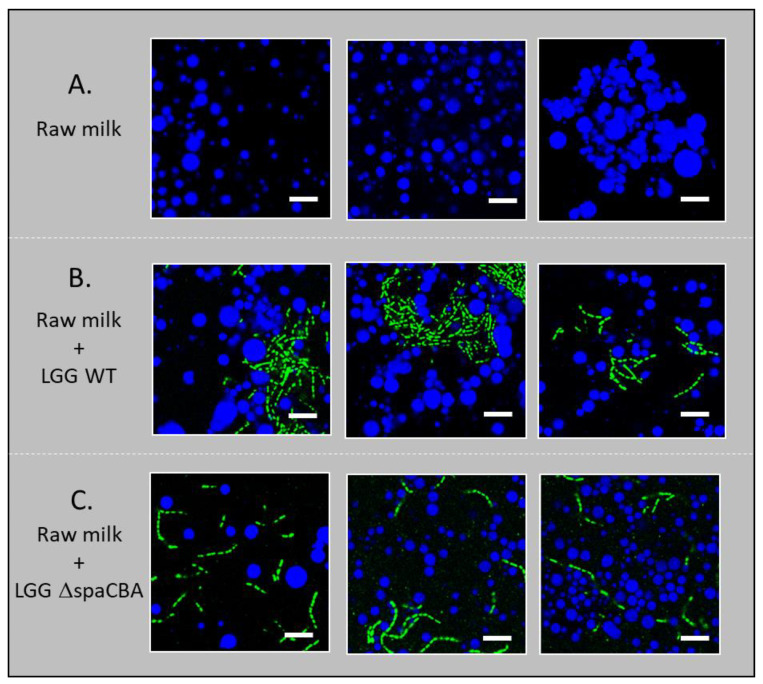
Confocal microscopy images: (**A**) raw milk, (**B**) raw milk with LGG WT and (**C**) raw milk with LGG Δ*spaCBA*. The fat globules are shown in blue and the bacteria in green. The scale bar represents 10 µm.

CLSM imaging was performed in order to investigate (i) how the presence or absence of bacteria might affect the distribution of milk fat globules and bacterial distribution itself, and (ii) how pili at the bacterial surface might affect the distribution of bacteria (single chains vs. aggregated) and their location in the dairy matrix (in the protein phase or in the vicinity of fat globules). Therefore, these images shed light on the mechanism of movement of these bacteria towards raw milk components. The images presented show both the bacteria-to-matrix organization and the bacteria-to-bacteria organization (Figure 3). First of all, it can be seen that, as expected, fat globules cover a large range of sizes (from ~1 to 10 µm). In raw milk, the LGG WT strain appears to be more aggregated than LGG Δ*spaCBA,* which was regularly distributed as single chains within the matrix. The cell aggregation of highly adhesive strains was previously observed for LGG and other lactic acid bacteria incorporated in whey solutions while they were homogenously distributed in a culture medium [30]. Overall, the CLSM results indicate that microbial surface properties may affect the location of the cells within the matrix. As the cells can be found in both aggregated (LGG WT) and individual (LGG Δ*spaCBA*) configurations, the impacts on emulsion stability may be totally different. Differences in microbial distribution could either be due to interactions of the bacteria with the milk components, i.e., through SpaCBA pili-β-lactoglobulin [23,31] and/or MFGM interactions [25], or to cell clustering, which would lead to increased size and more restricted movement through the sample. Therefore, without considering dynamic movements, bacteria might form a physical barrier to fat globule flocculation.

### 3.3. Matrix Structuration Dynamics

**Figure 4:**
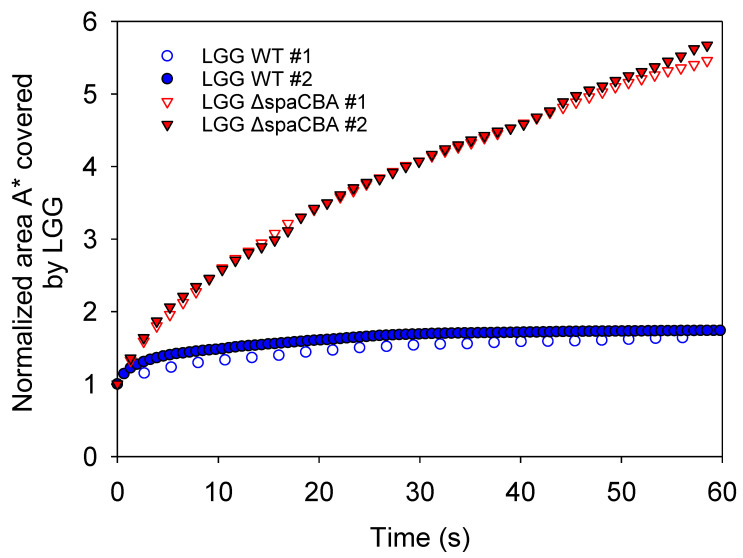
Normalized area (A*) covered by LGG for 60 s obtained from analysis of CLSM image sequences. Data were obtained in two independent samples with different acquisition frequencies.

Using image sequences from CLSM and the measurement of the cumulative area covered by bacteria over time ($A^{*}$), the different movement behaviours of the two strains were highlighted. For LGG WT, pili-milk component interactions allow bacteria to adhere to the surrounding matrix or to other bacteria. These interactions lead to the formation of large clusters of bacteria in the milk matrix and the entrapment of some of the fat globules. The cumulative area $A^{*}$ for this strain was plotted as a function of time (Figure 4). It can be observed that for LGG WT, the value of ($A^{*}$) remains close to the unity over time. This implies that the bacteria forming large clusters, but also individual chains, have no motility or diffusive motion in raw milk. The thermal effect is negligible for this size range. In the case of LGG Δ*spaCBA*, the pili-depleted strain, a homogenous distribution of bacteria in the milk matrix can be observed. The image sequences reveal erratic movements of the bacteria similar to Brownian motion. The cumulative area ($A^{*}$) for this strain increases continuously over time. This result shows the significant motion of the bacteria in the matrix and the possibility of steric interactions with fatty globules. As they do not express pili at their surface, LGG Δ*spaCBA* bacteria are unable to adhere to milk components such as MFGM [25] or β-Lactoglobulin [23,31]. Further experiments are necessary to discriminate between the processes inducing the motion of bacteria and the interactions involved.

## 4. Discussion and Conclusions

In the present study, the effect of the addition of bacteria (LGG WT and LGG Δ*spaCBA*) into raw milk was investigated. The microscopic observations can be related to the measurements of emulsion stability acquired using the Turbiscan and explaining the observed phenomena.

The large clusters formed by LGG WT entrapped some globules and could interact weakly with free globules, leading to a slight decline in the creaming rate and flocculation. The interaction of LGG WT with MFGM, through its SpaCBA pili, has been previously reported and could explain the trapping of some fat globules [25]. On the other hand, the bacterial aggregates might be mediated by the interaction of LGG WT, through SpaCBA, with β-lactoglobulin [31]. Indeed, LGG WT was found to be randomly distributed when analysed in culture medium while aggregated in whey protein solution [30]. *Lactobacillus aquaticus*, an LAB without predicted SpaCBA pili or a pili-like structure, exhibited a similar behaviour [30]. These observations confirm the key role of SpaCBA pili but preclude their exclusivity. Bacteria aggregates were also observed for the *Lactococcus lactis* MG1363 (pIL235pil) strain, which overexpressed pili, while the parental strain (MG1363) was homogeneously distributed in Gouda-type cheese [21] or fermented milk matrices [20]. The localisation of another strain of *Lactococcus lactis* in close proximity to fat globules, as well as proteins, was observed by transmission electron microscopy. The authors observed a correlation between fat content and the location of bacteria within Cheddar cheese with a different fat content [16,32].

The addition of LGG Δ*spaCBA* to raw milk led to a major effect. The bacterial aggregates were no longer observed, and bacteria were homogeneously distributed within the matrix, such as in culture media or whey protein solution [30]. Due to their dispersion and motion in raw milk, LGG Δ*spaCBA* bacteria were able to affect creaming by forming a barrier between fat globules, thereby delaying flocculation. A rough estimation of the number of bacteria (CFU) and fat globules indicates a ratio of 1/6. The probability of interactions is, therefore, high and may explain the strong impact on raw milk stability.

The present results highlight the crucial role of bacterial distribution and interactions with the surrounding matrix in helping to understand matrix structuration. In this study, the main mechanism of raw milk emulsion instability is attributed to flocculation rather than coalescence. LGG WT and the pili-depleted mutant have the ability to limit flocculation, which is the first step in the creaming phenomenon. The aim of using the wild-type and pili-depleted mutant was to determine differences in behaviour towards fat globules according to cell surface composition. By combining global measurements (emulsion instability) with microscopic observations, it was possible to describe how bacteria can impact matrix structuration. In the present case, LGG WT expressing pili at its surface was able to interact with the matrix components, but also with other bacteria, which led to the formation of a motionless network that slightly reduced the flocculation rate of the fat globules. On the other hand, the pili-depleted strain was distributed homogeneously within the matrix and displayed a motion that enabled it to occupy more space and limit the contact of fat globules. The structuration of dairy matrices may be strongly impacted by LAB, and in particular by molecular actors at their surface which modulate and contribute to their ability to adhere to the matrix components. The role of LGG in improving emulsion stability over time was evidenced in this study, which could be usefully extended to other LAB. The future work would consist of finding a correlation between the cell surface properties and the cell surface components of other LAB and their propensity to affect raw milk stability.

## Figures and Tables

**Table 1:** Emulsion stability parameters obtained for raw milk without LGG, and in the presence of either LGG WT or LGG Δ*spaCBA*.

	ΔBackscattering Intensity (%)	Creaming Thickness (mm)	Creaming Rate (µm/min)
Raw Milk	2.6	3.3	62.1
+LGG WT	1.1	2.8	29.6
+LGG *ΔspaCBA*	0.7	2.5	21.2

## Data Availability

Not applicable.

## Supplementary Materials

The following are available online at https://www.mdpi.com/article/10.3390/foods10050991/s1, Figure S1: Example of data obtained by monitoring emulsion stability (raw milk) over time using the Turbiscan. Figure S2: Control of particle variation in raw milk after a first analysis by Turbiscan and homogenization. The decline in the percentage of backscatter in the centre of the sample demonstrates the presence of flocculation and excludes the observation of coalescence in the sample. Figure S3: (A) Thickness of the cream layer obtained on the top of the sample as a function of time. (B) Backscattering intensity in the centre of the cylinder (35 mm) as a function of time. Raw milk (blue circle), raw milk-containing LGG WT (green circle) and raw milk-containing LGG ΔspaCBA (red triangle). Data obtained for a different raw milk batch.
