# Supplementary material for: Impact of Lacticaseibacillus rhamnosus GG on the Emulsion Stability of Raw Milk
Source: Foods. 2021 May 1;10(5):991. doi: 10.3390/foods10050991 (PMC8147333; doi:10.3390/foods10050991)
Supplement: Supplementary file 1 [file foods-10-00991-s001.zip › foods-1189899-SI.pdf]

foods

MDPI

Supplementary material:

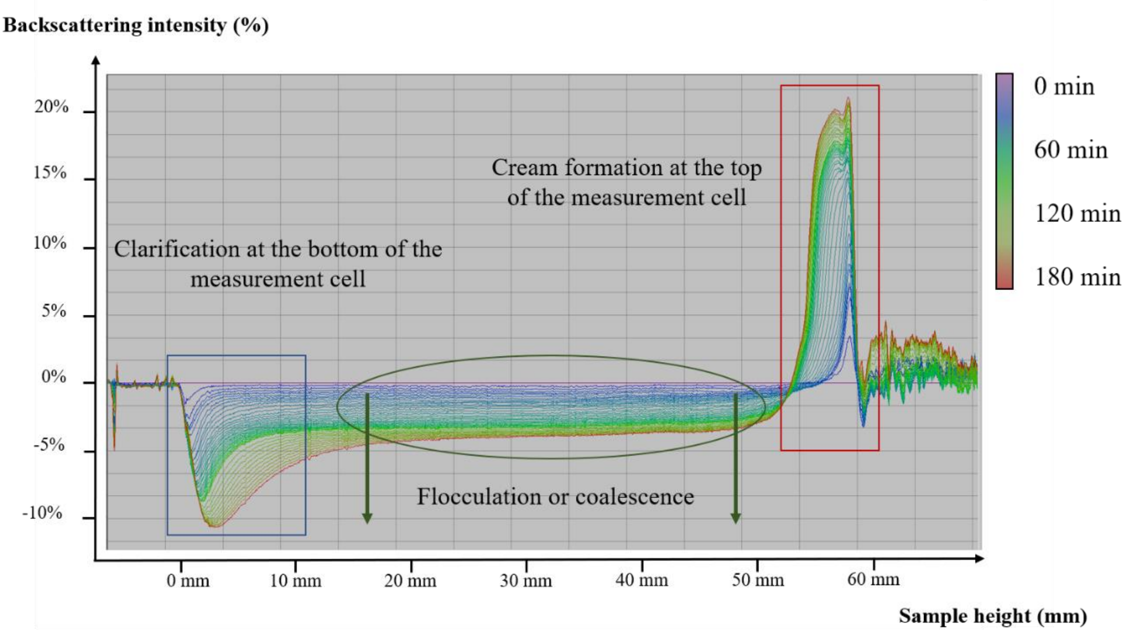

Figure S1: Example of data obtained by monitoring emulsion stability (raw milk) over time using the Turbiscan.

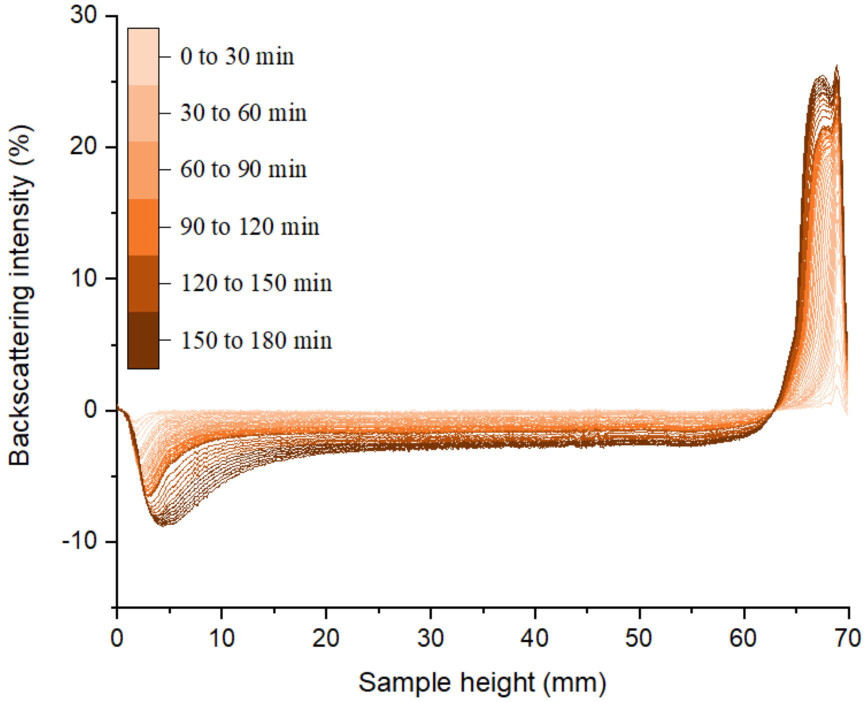

Figure S2: Control of particle variation in raw milk after a first analysis by Turbiscan and homogenization. The decline in the percentage of backscatter in the centre of the sample demonstrates the presence of flocculation and excludes the observation of coalescence in the sample.

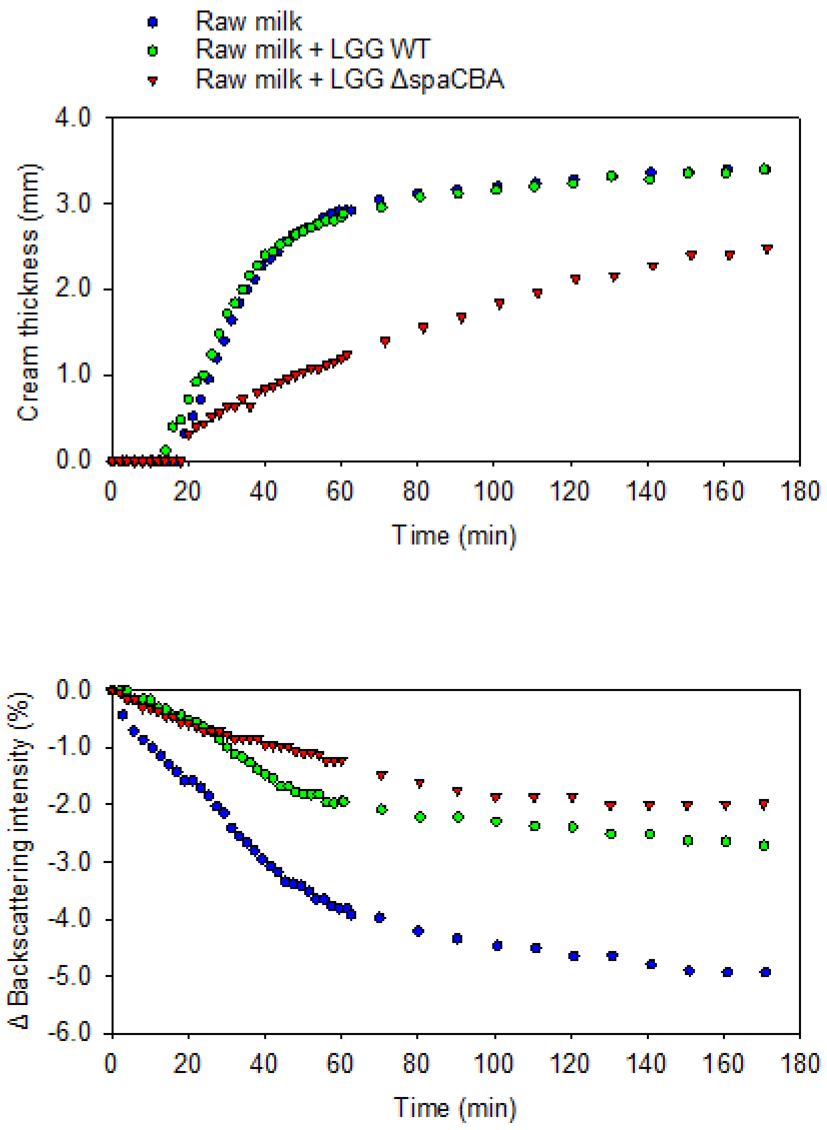

Figure S3: (A) Thickness of the cream layer obtained on the top of the sample as a function of time. (B) Backscattering intensity in the centre of the cylinder (35 mm) as a function of time. Raw milk (blue circle), raw milk-containing LGG WT (green circle) and raw milk-containing LGG ΔspaCBA (red triangle). Data obtained for a different raw milk batch.
